# The influence of length of implant on primary stability: An *in vitro* study using resonance frequency analysis

**DOI:** 10.4317/jced.53302

**Published:** 2017-01-01

**Authors:** Anwar B. Bataineh, Ala M. Al-dakes

**Affiliations:** 1 BDS, MScD, CSOS, MDSc, Professor Oral & Maxillofacial Surgery, Faculty of Dentistry, Jordan University of Science & Technology, Irbid, Jordan; 2 BDS, MScD, Lecturer of Oral & Maxillofacial Surgery, Faculty of Dentistry, Jordan University of Science & Technology, Irbid, Jordan

## Abstract

**Background:**

Primary stabilityis not sufficientin less contact area between the implant and bone, the healing process because will be disrupted due to micro-motions and fibrous tissue affects osseointegration.

**Material and Methods:**

We implemented an in vitro experimental study of total 135 XiVE® implants were inserted in 22.5 bovine cow ribs with bone quality similar to a type IV human bone. Each rib end received a group of three different implant lengths, which were 8mm, 13mm and 15mm and had the same diameter 3.8mm. Immediately after the implant placement, its primary stability was measured using Osstell Mentor equipment. ANOVA Tukey’s honest to test the significant difference were performed for data analysis between the resonance measures of the different lengths of implants. Statistical significance was assessed at a level *P*< 0.05.

**Results:**

A total of 45 implants were inserted for each length at cortical bone level. A significant difference between the three groups in favor of implant with 15mm length group (*P* = 0.000).

**Conclusions:**

Increasing dental implant length is considered to play a fundamental role in increasing dental implant primary stability, even in poor bone quality, through controlling the bone preparation process.

** Key words:**Dental implants, primary stability, resonance frequency analysis.

## Introduction

Osseointegrated dental implants have been accepted as one of the major treatment concepts for restoring completely and partially edentulous patients over the last three decades (1,2). Osseointegration depends on a variety of factors and inadequate control of these factors affects the stable anchorage of the implant to the bone tissue (3). One of the factors involved in the success of osseointegration and the long-term success of implants is the implant primary stability, which is defined as the biometric stability of the implant immediately after its placement within the bone (1,4,5). Primary implant stability is defined as the absence of mobility in axial, lateral, and rotational directions in the bone bed, immediately after insertion of the implant. It depends on the quantity and quality of bone, surgical technique, osteotomy size in relation to the implant diameter, and implant design, length, diameter, and type (3,4,6). Primary implant stability is the most important clinical goal to be achieved at the time of implant placement to define the best moment for implant loading (7).

The concepts of primary stability is considered essential to determine, because this can serve as a guide regarding the choice of treatment protocol; that is, immediate, early or delayed loading (4). Primary implant stability is related to the mechanical engagement of an implant with the surrounding bone after implant insertion. Secondary stability depends on bone formation and remodeling at the implant-bone interface, and is influenced by the implant surface and the wound-healing time (3,4,6).

The relationship between dental implant length and dental implant primary stability has been a controversial issue for many years (8,9). Different lengths of dental implant are generally available and range from 6mm to 20 mm. The most common implant lengths used in dentistry are between 8mm to 15mm, which resemble the natural root lengths. ˮStandard length implant’’ referred to the shortest implant length for predictable success to occur and was considered to be at least 10 mm (10).

Many studies suggested that increasing implant length plays an important role in decreasing the bone stress, and increasing implant stability in poor quality bone, such as bone type IV (11). Bone stress can occur at both the cortical and cancellous part of the bone. Increased implant diameter will lead to a decrease in bone stress in the cortical part of the bone, but increased dental implant length will decrease bone stress in the cancellous part of the bone. For the best combination of stress reduction in both bone types, implants that are 4.0 mm in diameter and 9.0 mm or more in length are considered the optimal implant to be selected in type IV bone. Meijer *et al.* (12) observed that the length of the implant had little effect on the amount of stress level, but the height of the mandible has a large influence on the amount of stress because of the overall deformation of the bone as a reaction to loading.

The purpose of this *in vitro* study was to investigate the influence of length of implant on primary stability in bone type IV based on resonance frequency analysis using Osstell Mentor test equipment.

## Material and Methods

This study was carried out in the Dental Teaching Center of Jordan University of Science and Technology. We used 22.5 fresh cow ribs of similar anatomical characteristics. In cross section, these bones are equivalent to a type IV human bone. All of the ribs came from the same cow. These ribs served as a model of a toothless human jaw, due to their macroscopic composition of cortical and medullary bone. Each bovine rib block was frozen for storage, then melted for 30 minutes in a water bath immediately before implantation. The ends of the ribs, of greater diameter, with a smaller cortical and a greater proportion of medullary bone, most closely resemble the type IV bone.

Three different implant lengths were used; 8mm, 13mm and 15mm; all implants had the same diameter (3.8mm). There were 135 XiVE® implants (DENTSPLY Friadent, Mannheim, Germany) inserted in this study; 45 of each of the 3 lengths. The XiVE® implant system has good clinical effectiveness in edentulous patients. It has good biocompatibility and osseointegration, and its shape was designed to resemble the root of the natural tooth. It presents a grit-blasted and acid-etched implant surface. The thread design for XiVE® implant is unique in its shape. In the crestal region (cortical bone area) the thread profile has flat area with low cutting resistance to prevent pressure necrosis, which could be caused due to excessive compression. In the cancellous bone, the implant has a narrow thread profile with a deep thread, which is recommended for adequate primary stability.

**Figure 1:**
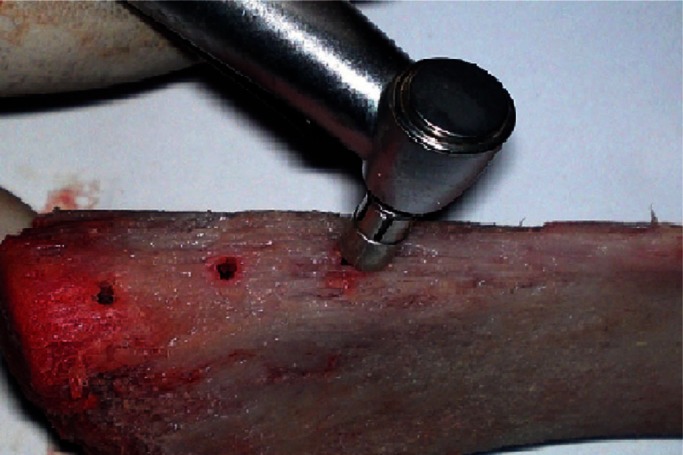
Preparation of the three implant beds with 7 millimeters inter-implant distance.

Six implant beds were prepared in each rib block, three on each end of each rib. Each preparation was made according to the manufacturer’s protocol. Preparations corresponded to different implant lengths and routine implant bed preparation (drilling technique) was used. Standard drilling protocol as recommended by the manufacturer was completed, as was elimination of excess cortical bone by the use of a countersink. To prevent the movement of the ribs during the preparation and measurements of the implant bed, the cow ribs were fixed firmly on the table using a specific handle. Each test bed should have at least 5 millimeters of bone around it. Therefore, an inter-implant distance of 7 millimeters was maintained (Fig. 1).

**Figure 2:**
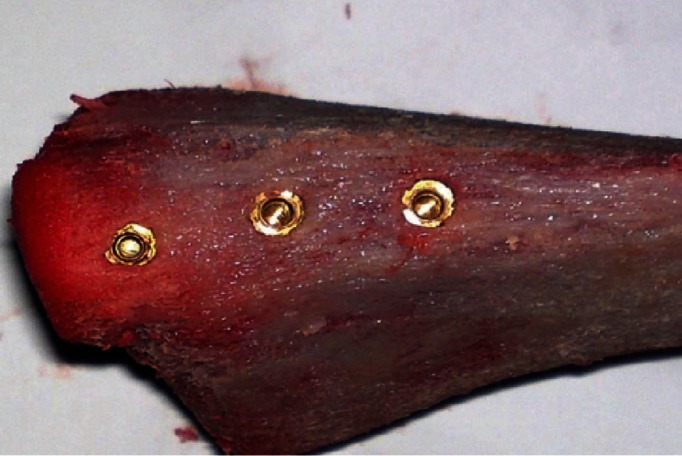
The three dental implants in each preparation bed.

The implant preparation was almost symmetrical in all implant preparation. Caution was taken not to overheat the bone during the drilling process, as that can result in bone cell death. To avoid the potential for overheating, the bone drills used were sharp and were not used in a manner where excessive drill speed or pressure was involved. Saline was used for continuous irrigation of the implant site to minimize the amount of heat generated. At the end of the drilling process, an alignment pin was placed to confirm that the hole created met the needed alignment and depth requirements for the dental implant that was placed. After confirming the implant bed preparation, the implant was screwed into the implant bed to cover the rough area, using its own implant system screwing instruments (Fig. 2). The implants were seated in such a way as to completely cover the rough area. Then the transducer corresponding to each length of implant was inserted, pressing them down manually.

**Figure 3:**
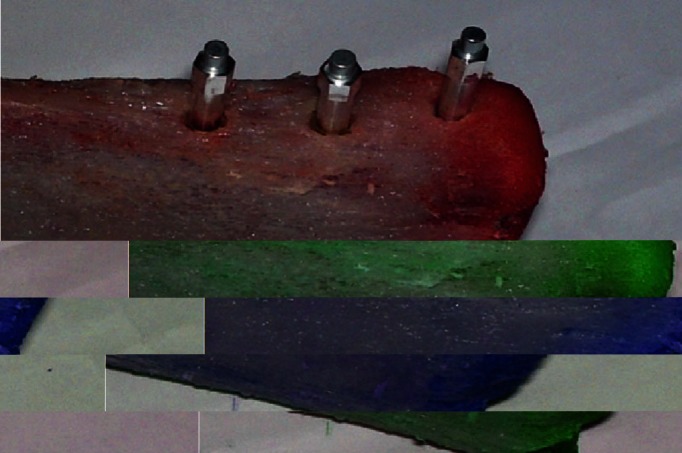
Dental implants after insertion the Smartpeg.

Implant primary stability were measured using Resonance Frequency Analysis (RFA) using the Osstell Mentor test equipment (Osstell™ mentor; Integration Diagnostics AB, Sweden) and Implant Stability Quotient (ISQ) index was also recorded. The ISQ is recorded as a number between 1 and 100, with 100 representing the highest degree of stability. The method involves the use of a small transducer (Smartpeg) that is attached to the implant (Fig. 3). The SmartPeg was handled carefully, as damages to the SmartPeg may affect the measurement result. The SmartPeg is magnetic, and the Mount will hold the SmartPeg as it is carried to the implant. The SmartPeg was screwed onto the implant using approximately 4-6 Newton centimeters (Ncm) of torque. It is important not to over tighten the SmartPeg to avoid destroying the SmartPeg’s threads. The SmartPeg is disposable, but may be used several times.

Then measurement of ISQindex with Osstell mentor, the probe was close to the top with a 90º angle to the transducer of the SmartPeg without touching it and the Osstell produced an audible tone. If two such sounds were heard in a row, they will be followed by a beeping sound and the display will present one or two ISQ values. Of the four measures obtained for each of the 135 implants (45 of each length of 8mm, 13mm, & 15mm), only the highest value for each implant was recorded. All of the implants were inserted mono-cortically, and clinically mobile implants were not included, due to the increased variability in the ISQ value, as recommended by the manufacturer.

Data analyses were carried out using the Statistical Software Package for Social Science (SPSS®)/13.0 (SPSS Inc. ®, Chicago, USA). Normal distribution analysis was performed to determine the type of analysis will be applied on the entered data. Statistical analysis using Univariant analysis of variance (ANOVA) was performed to test the statistical differences between the Resonance Frequency Analysis measures of the different lengths of implants. T-test and Tukey’s honest significant difference was used to report the difference between ISQ values and tests whether each length is significant. Statistical significance was defined at *P*< 0.05.

## Results

The overall study sample consisted of 135 XiVE® implants with three different implant lengths (8mm, 13mm and 15m) and had the same diameter (3.8mm) were used. A total of 45 implants were inserted for each length at cortical bone level. The highest ISQ value was recorded for each implant.

Kurtosis and Skweness tests were used to test the normal distribution of results. If the Kurtosis results ranged from -1.5 to 1.5 and Skweness from -1 to 1 then the readings were considered normally distributed, and this made it possible to apply the parametric tests for the results. The analysis results show that the value for Kurtosis was -1.50 and Skewness was -0.014. This indicates the recorded figures were normally distributed. Therefore, parametric analysis was applied to test the statistical differences for stability of the three treatments.

 Table 1 shows a comparison between the ISQ mean values of the implant length 8mm, 13mm and 15mm inserted at the cortical bone level using the conventional drilling technique. ISQ values of the dental implants of length 15mm were higher than both the 8mm and 13mm group. Statistical analysis using one-way analysis of variance (ANOVA) showed a significant difference between the three groups in favor of implant with 15mm length group (*P* = 0.0001).  Table 2 shows the results of the ANOVA for the different groups was significant, which indicates that there are significant differences among means.

**Table 1:**
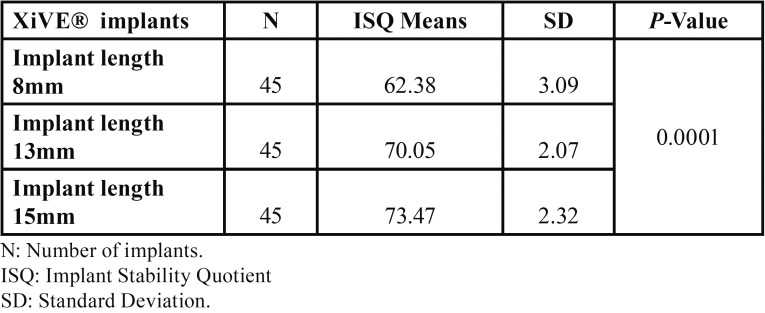
ISQ Values of XiVE® Implants having three different Lengths (8mm, 13mm, 15mm) Inserted Using Conventional Drilling Techniques.

**Table 2:**
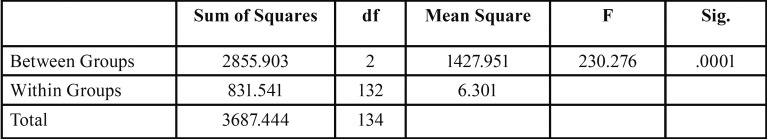
Analysis of variance for the different treatments.

Tukey’s test reports the difference between every possible pair of factor levels and tests whether each is significant. It also includes the boundaries for a 95% confidence interval around the size of each difference. Tukey’s test in this study indicates that there is a significant difference between 13mm length implants compared to 8mm group at (*P* ≤ 0.05). Similarly, the results showed significant difference for the 15mm length implants treatment from 8mm group. Additionally, there is a significant difference between 15mm length implants compared to 13mm group. The 15mm length is significantly more stable than the 13mm length, and the 13mm length is significantly more stable than the 8mm length, based on resonance frequencies ( Table 3).

**Table 3:**
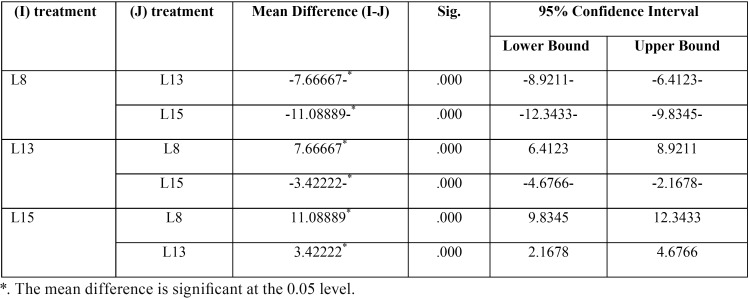
Multiple Comparisons using Tukey’s test.

## Discussion

Primary implant stability is the most important clinical goal to be achieved at the time of implant placement. Such stability contributes to determining the best moment for implant loading and increasing implant success rate (7).

The success of dental implant depends on both endogenous and exogenous factors. Bone quality is among the endogenous factors, and implant design is among the exogenous factors (13). For many years, implant configuration has been considered an essential requirement for implant success. Among the related implant parameters, diameter and length play key roles in implant success, since they directly influence the primary stability and removal torque values.

The effects of the dental implants’ length on their short and long term prognosis on primary stability has been a controversial issue (8,9). Several investigators showed clearly that short implants failed more often than longer implants (9,14,15). However, others reported that implant length did not appear to significantly influence implant survival rate (8,9). Several factors have been suggested to explain this, such as the implant’s primary stability and the quality of bone. In addition, it should be noted that some of the studies that reported lower survival rates with short implants used a routine surgical protocol independent of bone density (16). In 2006 Misch *et al.* (17) studied the failure rate of 2837 short implants (less than 10mm in length) which were placed in the posterior region in the mandible, to find the connection rationale between the high failure rates of posterior-placed short dental implants with the hypothesis that implant length does not influence success rates and they reported that the survival rate was 85.2 %.

On the other hand, increased crown height, high bite forces, and bone density are factors that affect the implant-bone interface and not the implant length. This is why posterior sites are not suitable for immediate loading (18). Renouard *et al.* (19) tried to explore the high failure rates of short implants, revealed that the surgical protocol used for short implant insertion did not include factors such as the evaluation of the bone quality and the implant surface. Miyamoto *et al.* (16) found that implant stability at the time of surgery might largely depend on local bone conditions rather implant length. Also, there are studies suggesting that the implant length cannot be considered in isolation, but only in conjunction with the height of the mandibular cross-section when considering the effect on strain concentration around an implant (20,21).

As far as concerns the impact of implant length on immediate loading protocols, the results indicate the use of implants longer than 10 mm. In a controlled study (22)reported 10 failed implants in the experimental group (immediate loading), while the control group (conventionally delayed loading) produced only one failure when inserting implants of various lengths (10-15 mm) with a standard diameter (3.75 mm, 4.5 mm). No statistical significant correlation was found between length and the cumulative survival rates, while the failures were significantly correlated with the insertion torque.

In regard to the analysis of stress on the implant, it should be noted that the bone-implant interface is an area of great importance for implant survival and success. Also, the significance of the implant-abutment interface has an important role for the vitality of implant-supported superstructure. Applied loading develops a highly deformed state at implant-abutment interface. Exceeding the proportional limit due to stress concentration may lead to joint opening (23). Many factors contribute to the mechanical integration at the implant-abutment interface, although there is no study concerning the effect of length on the stress field in the implant (24,25).

Many studies suggested that increasing implant length plays an important role in decreasing the bone stress and increasing implant stability in poor quality bone such as bone type IV (11). This might be expected because holding power is directly proportional to the amount of thread engagement (26). Andrés-García R *et al.* (27) founded that for dental implant to succeed it should be at least 10mm in the mandible and 13mm in the maxilla.

Many studies reported that the increased bone density will result in a high implant success rates. Thus implants placed in the posterior region of the maxilla, where bone density is low, had inferior success rate compared to that placed into the anterior mandible, where bone density is frequently higher (1,2,16). Many different studies in the literature corroborate lower success rates of implants placed in type IV bone (2,13). According to what has been established in other experimental studies which used cow ribs as a study model, the most distal region of the rib, which is of lower diameter and contains a lower proportion of cortical bone and greater proportion of medullary bone, would be similar to a type IV bone (28,29).

The Osstell® model used in the study was easy to use and handle; however, when obtaining values, one faces the problem that keeping the probe perpendicular to the transducer- different values are obtained according to the position on the horizontal plane in which it is placed. These differences in the values obtained are explained by the manufacturer as the values of “higher” or “lower” stability shown by the implant; terms not valued in literature. Therefore, we chose the higher value obtained in each test, each of them carried out at 90º degrees of separation between them and another one on the horizontal plane.

The objective of this research was to investigate the effect of implant length on implant primary stability. This research has demonstrated that increasing the implant length enhances the dental implant primary stability. The increase of implant length up to 15mm would increase the ISQ mean to 73.47, which is significantly more than that implant length 8mm and 13mm. This is consistence with the results indicated that increasing dental implant length is considered to play a fundamental role in increasing dental implant primary stability (14,18). Shorter dental implants, in comparison to longer dental implants, have a lower success rates due to small surface area and decreased crown-to-implant ratio. This will lead to an increase of stress at both implant and crestal bone. Furthermore, short dental implants will not dissipate all occlusal forces away from bone-implant interface (14). Reduced surface area of short implants at the bone-implant level will lead to a decrease in area of osseointegration and an increase in stress at the crestal bone, which can cause bone resorption.

Miyamoto *et al.* (16) studied the success rate for short and long implants. They found that when the implant diameter and position were kept constant, and implant length is the only variable factor, the success rate for short implants is 90.5 %, compared to 96.3 % for the longer implants.

Bovine rib did not achieve all the desired bone qualities in this study. However, we must not forget that this has been an experimental study carried out on an animal model, which involves a series of limitations, such as the quality of bone in an area other than the oral cavity, as well as lack in vascularization. Additionally, caution should be used to keep the probe perpendicular to the transducer. If not, this may lead to different values, according to the position on the horizontal plane in which it is placed. This study was able to confirm that longer dental implants showed an increase in primary stability over their than shorter counterparts. Therefore, long dental implants provided more primary stability than short ones, even in poor quality bone. It is important to note that the bone preparation process was strictly controlled. The validity of our results has been the subject of a solid statistical study, the results of which support our initial hypothesis.

The results of this study suggest that the increasing dental implant length plays a fundamental role in increasing dental implant primary stability. This study revealed the need to prepare new or additional lines of research in order to answer the possible questions that arise as a result of our.
